# Influence of Heat Sink on the Mold Temperature of Gypsum Mold Used in Injection Molding

**DOI:** 10.3390/polym12030701

**Published:** 2020-03-21

**Authors:** Chung-Chih Lin, Kun-Chen Chen, Hon-Chih Yeh

**Affiliations:** Department of Mechanical and Computer-Aided Engineering, National Formosa University, No.64, Wen-Hwa Road, Hu-Wei town Yunlin county 63208, Taiwan; smhsu@nhu.edu.tw (K.-C.C.); linccnfu@gmail.com (H.-C.Y.)

**Keywords:** injection molding, gypsum mold, mold temperature, heat sink, thermal circuit

## Abstract

Gypsum molds have been developed as an alternative for the Rapid tooling (RT) method used in injection molding. However, the poor capability of the heat delivery forces the gypsum mold to operate under a high-risk condition, and distortion of the molded part becomes apparent. The goal is to investigate the effect of a heat sink on the reduction of the gypsum mold temperature and to establish a methodology for the heat sink design. The methodology used the advantage of the electrical circuit concept to analyze the mold temperature. The heat transfer of a mold was modeled using an equivalent thermal circuit. After all the components on the circuit were determined, the heat transfer rate could then be calculated. Once the heat transfer rate was known, the mold temperature could be easily analyzed. A modified thermal circuit considering transverse heat conduction was also proposed, which estimated the mold temperature more accurately. The mold temperature was reduced by 16.8 °C when a gypsum mold was installed with a 40 mm thick heat sink in a parallel configuration. Moreover, the reduction of the mold temperature improved the deflection of the molded part from 0.78 mm to 0.54 mm. This work provides a quick approach to analyze the mold temperature based on the thermal circuit concept. As the cooling system of the mold was modularized analytically, important properties of the cooling system in the heat transfer process were revealed by analyzing the thermal circuit of the mold, for example, the heat transfer rate or the mold temperature.

## 1. Introduction

**Figure 1:**
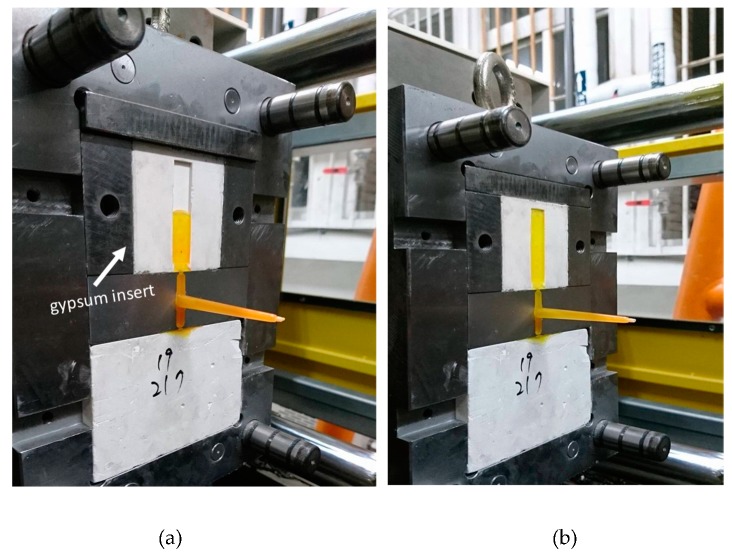
(**a**) The gypsum insert with the injection part at 50% of injection volume, and (**b**) the gypsum insert with the injection part at 100% of injection volume.

When a customer requests the development of a new product, a small quantity of the prototype is usually prepared for validation. Rapid tooling (RT) methods provide a quick way to satisfy this requirement. In general, the volume shrinkage of most polymer-based RT materials occurs when the material state changes from a melted state to a solid state. The volume of a gypsum block expands after the gypsum paste becomes solidified and the gypsum mold does not suffer from the volume shrinkage that is a known processing issue for many polymer-based RT materials. This specific material property improves the replication capability. The excellent replication capability of microstructures makes gypsum molds good candidates for precision molding, for example, gypsum molds are frequently used in denture application. Gypsum (calcium sulfate dihydrate) is a well-known mineral obtained from natural resources. However, gypsum molds are often used in low-pressure molding, e.g., casting [1,2,3], due to the weak mechanical strength of gypsum material. In our previous work [1], we developed gypsum inserts and gypsum molds for the RT method to be used in plastic injection molding. Since gypsum is a natural material and can be easily recycled, gypsum molds provide an ecofriendly approach for producing a limited quantity of prototypes made by using thermoplastic materials, with the injection molding method as the intended production line. Figure 1a,b show examples of parts molded using a gypsum mold in plastic injection molding.

Injection molding is a sequential manufacturing process that produces products one by one periodically. The resin is heated and transformed into molten polymer, and then, the injection pressure and speed force the molten polymer into a mold cavity. As the molten polymer continues to cool and becomes strong enough, the mold opens and the cooled product is then stripped from the cavity. The qualities of the molded product, such as dimensional stability, appearance, and mechanical properties, are highly dependent on the processing conditions. One of the most critical variables determining the final quality of the product is the mold temperature [2,3,4,5,6]. The mold temperature is dynamic during the injection molding process. To lessen the mold temperature variation, the mold is usually designed with a cooling system to keep the mold temperature as constant as possible. An unsuitable mold temperature not only influences the quality of the molded product, but it may also increase production costs [7,8,9]. An RT mold is used only for a small number of productions and the problem resulting from the mold temperature in the injection process is often neglected. Nevertheless, Segal and Campbell [10] pointed out that engineers making use of the RT method to produce technical prototypes must be aware of the effect of the mold temperature on the properties of the final part. Mendible et al. [11] evaluated three different inserts: (PolyJet) 3D printing using digital ABS, direct metal laser sintering (DMLS) using bronze, and machining using stainless steel. Without a cooling system in the inserts, they found that the shrinkage value of the molded part using PolyJet was the largest. Wu et al. [12] proposed the optimization of the conformal cooling design for a uniform mold temperature of the RT mold. Hopkinson and Dickens [13] tried to predict the ejection force and the tool lifetime of an RT mold because of their poor heat transfer characteristics. Aluru et al. [14] also studied the effect of the thermal conductivity of RT molds on final-part distortions using a simulation tool. They compared the molding performance of the RT mold and the conventional steel mold using simulation results and actual molding experiments. All of their studies indicated that the cooling system within the mold insert was important, and its effect should be evaluated in the application of the RT mold. In addition, they pointed out that an analytical approach was still required. How to analyze the design parameters of a cooling system, such as the location or the diameter of the channel, still need to be addressed.

In general, the design of the cooling system is usually implemented by the designers’ experiences or the use of commercial software, which may be time-consuming in respect to computation and the lack of the physical essence of an active system. Pignon et al. [15] provided an analytical approach for choosing thermal conditions during thermoplastic injection molding. The proposed models allowed quick determinations of the cooling/solidifying time, the mold surface temperature variation, and the heat flux densities exchanged between the polymer and the mold. Zarkadas and Xanthos [16] developed a simplified semi-analytical method to predict the cooling time of the part in an injection molding process. Their study demonstrated that the determination of an engineering problem can be more efficient if an analytical model can be established in advance. 

Materials used in RT methods are characterized by low thermal conductivity, and this represents an issue of the thermal effect for processing. A coolant system can improve the heat transfer capability but increases the cost and complexity as well. In our work, we introduced a heat sink made of aluminum as the cooling system when forming the gypsum inserts and, thus, improved the heat transfer capability. We also developed a methodology based on the thermal circuit concept [17] to easily determine the design parameters of the heat sink, such as the thickness and installation configuration (e.g., series or parallel) of the heat sink. The influences of the design parameters on the gypsum mold temperature were investigated with thermal circuits and validated by experiments. In addition, a modified thermal circuit considering transverse heat conduction was proposed, which extended a 1D model to a 2D heat transfer. It provided a more accurate model to predict the mold temperature. Finally, the influence of the mold temperature on the deflection of the molded parts was also investigated in this work.

## 2. Formulation to Analyze Mold Temperature

When the shape of the molded part was symmetrical and the mold cavity was located in the center of the mold, the heat transfer process inside the mold was assumed to be symmetrical and could be modeled using the 1D heat transfer equation [17]. Heat convection can be neglected due to the very high viscosity of the molten polymer (more than 1000 Pa·s), and the molded part was cooled mainly by conduction [18]. Two mold halves (the fixed side and the mobile side) constructed the mold cavity, and the heat transfer happened on each side of the mold insert. Suppose the dimensions of the mold insert for both mold halves were the same, and the heat transfer process in each mold insert was regarded identically. The heat conduction started from the surface of the insert, and the heat flow direction was assumed to be normal to this surface. All of the thermal properties, such as thermal conductivity of the gypsum and heat sink, were considered constant during the heat diffusion process. The 1D heat transfer equation, through the thickness of the mold insert in the *x*-direction, was given by [15]:

(1) $$\frac{\partial }{\partial x}\left(k\frac{\partial T}{\partial x}\right)=\rho_{m}C_{m}\frac{\partial T}{\partial t}$$

where *k*, *ρ_m_*, and *C_m_* are the thermal conductivity, density, and specific heat capacity of the mold insert material, respectively. At the initial time of the injection process, the mold cavity was instantaneously filled, and the temperature field on the mold cavity surface was assumed to be uniform and equal to the melting temperature *T_p_*. The initial condition was defined by:

(2) $$T\left(x,\;t=0\right)=T_{p}$$

In addition, since the heat transfer equation is second order in the spatial coordinate, two boundary conditions need to be specified to determine the temperature distribution in the mold insert. Heat flux densities, *φ*_1_(*t*) and *φ*_2_(*t*), were imposed on the top and bottom surfaces of the mold insert. They were expressed by:

(3) $$-k\frac{\partial T\left(x,t\right)}{\partial x}=\varphi_{1}\left(t\right)\;\;\mathrm{at}\;x=0$$

(4) $$-k\frac{\partial T\left(x,t\right)}{\partial x}=\varphi_{2}\left(t\right)\;\mathrm{at}\;x=h$$

in which *h* represents the thickness of the mold insert. Both of them were to be determined using the experimental data. Hence, the temperature *T* could be calculated by using the finite difference method [19]. 

At the beginning of the injection process, the mold temperature rises gradually and then achieves a plateau with the increase in the injection molding cycle. In this stage, the mold temperature is supposed to be in a periodic stable state. A semi-empirical equation based on the assumption of a linear evolution of the mold temperature [15] provides an efficient way to determine the cavity temperature, *T*_s_, in the steady state. *T*_s_ was determined by:

(5) $$T_{s}=1/2\left(T_{c}+T_{mi}\right)$$

where *T*_c_ was the thermal contact temperature as the molten polymer contacted the cavity’s surface at the end of the filling stage, and *T_mi_* represented the mold temperature before injection molding. Note that *T*_c_ can be calculated from Equation (1).

For a steady-state condition with no extra source or sink of energy within the wall, the heat flux density is a constant and independent of a transfer path [17]. The appropriate form of the heat equation is given by:

(6) $$\frac{d}{dx}\left(k\frac{dT}{dx}\right)=0$$

**Figure 2:**
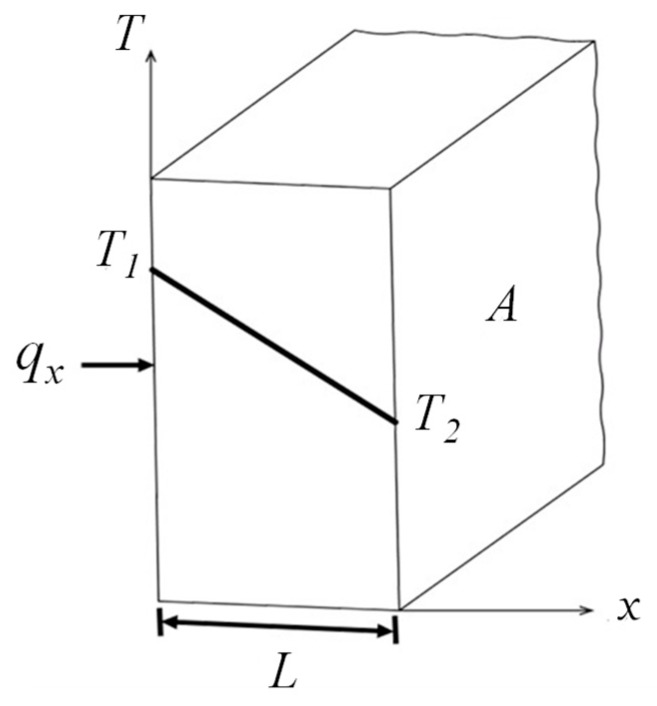
1D heat transfer through a plane wall.

Fourier’s law implies that the 1D heat transfer rate, *q_x_*, is directly proportional to the temperature difference, Δ*T* = (*T*_1_ − *T*_2_), and cross-sectional area *A*, as well as varying inversely with Δ*x*. Therefore, for 1D steady-state conduction in a plane wall with no heat generation, the temperature varies linearly with *x*, as shown in Figure 2. The simplified heat transfer rate equation of the wall can be expressed by:

(7) $$q_{x}=kA\frac{\Delta T}{\Delta x}=kA\frac{T_{1-}T_{2}}{L}$$

### 2.1. Thermal Resistance

The concept of thermal resistance is referred to as the electrical circuit. There exists an analogy between the diffusion of heat and the movement of electrical charge. Just as an electrical resistance is associated with the conduction of electricity, a thermal resistance may be associated with the conduction of heat. The resistance is defined by the ratio of the driving potential to the corresponding transfer rate [20]. Therefore, the thermal resistance for conduction *R* can be derived from Equation (7) and defined ideally by the ratio of the temperature difference Δ*T* to the heat transfer rate *q_x_*. In fact, any material defects, such as a small void or non-uninform structure resulting from manufacture process, may influence the accuracy of the thermal resistance. A modified thermal resistance with a correction factor is proposed and defined by:

(8) $$R=\left(c_{1}\frac{x}{\Delta x}+c_{0}\right)\frac{\Delta x}{kA}$$

where *x* is the measurement location, and the coefficients *c*_1_ and *c*_0_ can be determined by the experimental data. 

Similarly, a thermal resistance may also be associated with heat transfer by convection at a surface. The thermal resistance for convection is then given by:

(9) $$R_{conv}=\frac{1}{h_{cv}A}$$

where *h_cv_* is the convection heat transfer coefficient.

When a gypsum insert is installed with a heat sink for heat transfer improvement, it can be regarded as an assembly of different layers of materials. Therefore, the heat diffusion of a gypsum insert can be modeled using several thermal resistances in a thermal circuit. As the equivalent thermal resistance of the circuit is determined, the heat transfer rate can be calculated, and then, the temperature on each layer of the insert can be calculated analytically.

### 2.2. A Series Configuration of the Composite Wall

**Figure 3:**
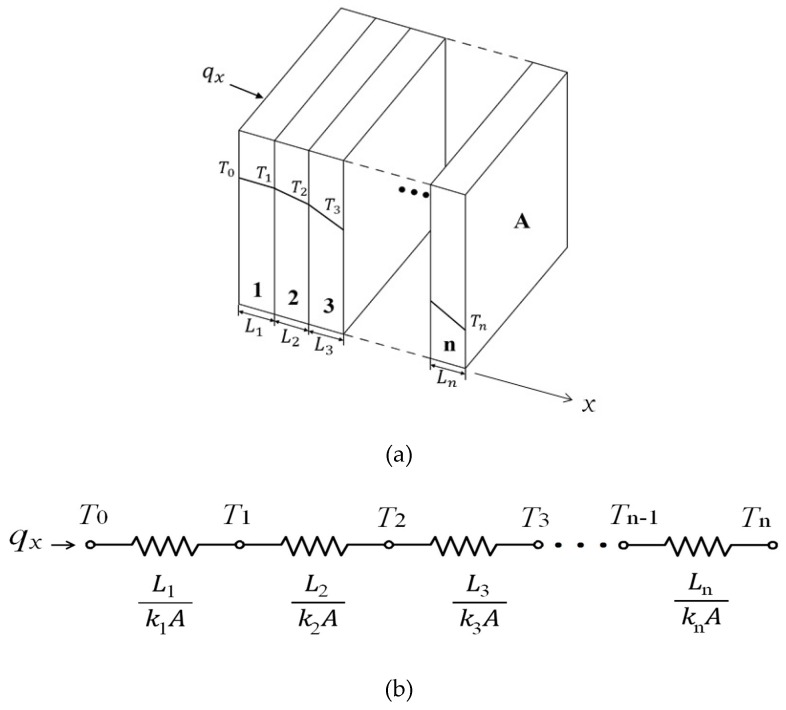
(**a**) A series configuration of the composite wall; (**b**) the thermal circuit of the composite wall.

Suppose that *n* pieces of wall have thicknesses and thermal conductivity of *L*_i_, and *k_i_*, *i* = 1, 2, …, *n*, respectively, and are assembled in a series configuration, as shown in Figure 3a. Assume that the material of each piece of wall is uniform, and each adjacent wall comes into contact with each other perfectly. Hence, the heat loss between their interfaces can be neglected. The heat is conducted from the first wall to the last wall, sequentially. The thermal circuit of the composite wall can be sketched using Figure 3b. The node in the circuit represents the temperature of the location. In accordance with the electrical circuit concept for a series configuration, the heat transfer rate, *q_x_*, may be determined by separate consideration of each element in the circuit. That is:

(10) $$q_{x}=\frac{T_{0}-T_{1}}{L_{1}/k_{1}A}=\frac{T_{1}-T_{2}}{L_{2}/k_{2}A}=\cdots =\frac{T_{n-1}-T_{n}}{L_{n}/k_{n}A}$$

where the nodes in the circuit, *T*_0_, *T*_1_, …, and *T_n_* are the surface temperatures for each layer. The equivalent thermal resistance of the thermal circuit, $R_{e}^{S}$, is the sum of the resistances of each wall and is expressed by:

(11) $$R_{e}^{S}=\frac{1}{A}\left(\frac{L_{1}}{k_{1}}+\frac{L_{2}}{k_{2}}+\cdots +\frac{L_{n}}{k_{n}}\right)$$

In terms of overall temperature difference, *T*_0_-*T_n_*, the heat transfer rate is determined by:

(12) $$q_{x}=\frac{T_{0}-T_{n}}{R_{e}^{S}}$$

### 2.3. A Parallel Configuration of the Composite Wall

**Figure 4:**
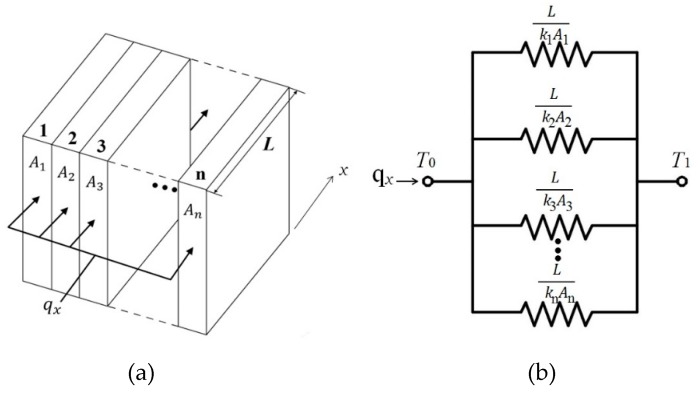
(**a**) A parallel configuration of the composite wall; (**b**) an equivalent thermal circuit of the composite wall.

When the walls are characterized by a parallel configuration, as shown in Figure 4a, heat transfer occurs simultaneously on the contact surfaces of all the walls. The heat flow is now two-dimensional; however, it is often reasonable to assume one-dimensional conditions as the thickness of the heat sink is small [17]. The thermal circuit can be sketched as shown in Figure 4b. All of the surfaces normal to the *x*-direction where the heat is delivered are assumed to be the same temperature. Therefore, the equivalent thermal resistance of the thermal circuit, $R_{e}^{P}$, is calculated by:

(13) $$R_{e}^{P}={\left(\frac{k_{1}A_{1}}{L}+\frac{k_{2}A_{2}}{L}+\frac{k_{3}A_{3}}{L}+\cdots +\frac{k_{n}A_{n}}{L}\right)}^{-1}$$

### 2.4. Thermal Circuit of the Gypsum Insert

**Figure 5:**
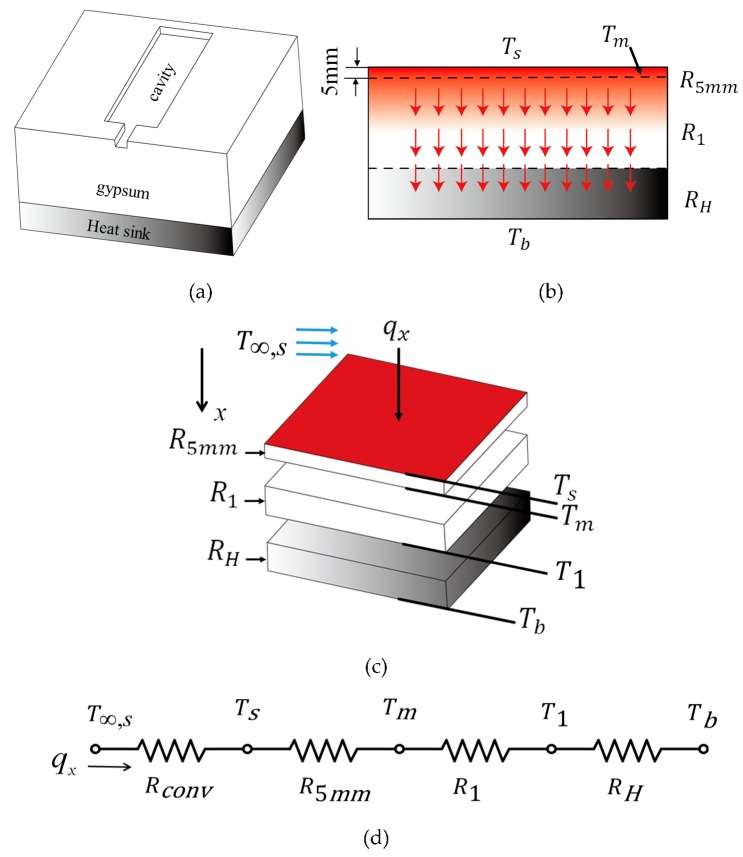
(**a**) A heat sink installed in the gypsum insert with a series configuration, (**b**) heat diffusion in the central cross-sectional view, (**c**) a sketch of the analysis model, and (**d**) the corresponding thermal circuit.

When molten polymer is injected into the mold, the duration of the filling stage is usually very short, and the whole temperature on the mold cavity can be assumed to be the same during this stage. Suppose the mold cavity is located in the center of the gypsum insert with a heat sink, as shown in Figure 5a. The heat conduction starts from the top of the gypsum insert and passes through different layers, such as the gypsum layer and heat sink and the bottom of the gypsum insert, sequentially. The heat diffusion process in the central cross-sectional plane of the insert can be referred to in Figure 5b. The mold temperature, *T_m_*, is defined by the temperature measured at a depth of 5 mm below the cavity’s surface of the gypsum insert [21,22]. *T_s_*, and *T_b_* stand for the cavity temperature and the bottom temperatures of the gypsum insert, respectively. *T_∞,s_* represents the room air temperatures on top surface of the insert. The discrete components of the insert and its thermal circuit are constructed as shown in Figure 5c,d, respectively. For a series configuration of the gypsum insert, the equivalent resistance, $R_{e}^{s},$ of this circuit can be calculated by:

(14) $$R_{e}^{s}=\left(R_{5mm}+R_{1}+R_{H}+R_{conv}\right)$$

where *R_5mm_*, *R_1_*, and *R_H_*, represent the thermal resistances of each layer where the heat is conducted. *R_conv_* is the thermal resistance for environmental convection. 

**Figure 6:**
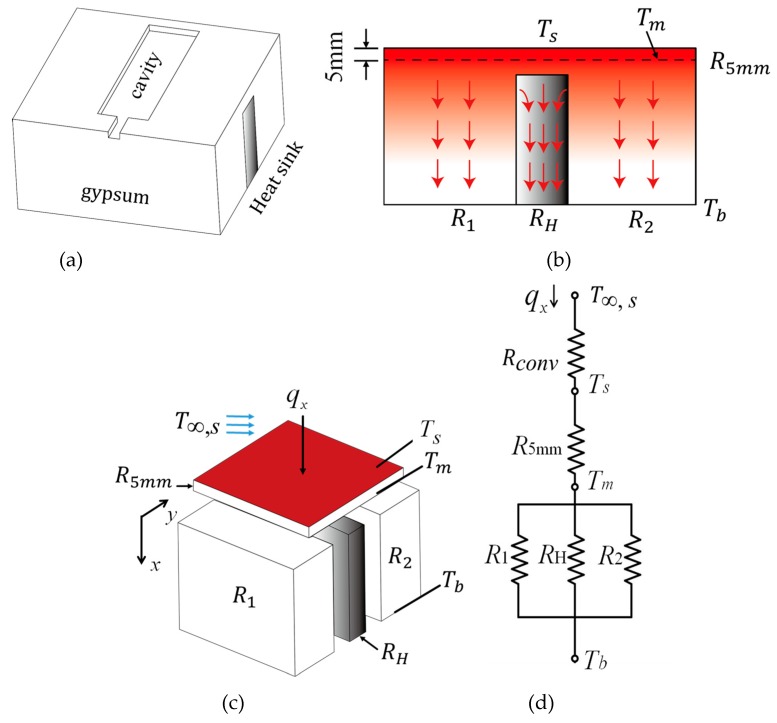
(**a**) A heat sink installed in the gypsum insert with a parallel configuration, (**b**) heat diffusion in the central cross-sectional view, (**c**) a sketch of the analysis model, and (**d**) the corresponding thermal circuit.

Similarly, if a heat sink is installed into the gypsum insert in accordance with Figure 6a, a sketch of the heat conduction process is illustrated in Figure 6b. The heat in the cavity is first conducted through the top layer, and then, the heat passes through both the gypsum layer and the heat sink simultaneously. The discrete components of the insert and the corresponding thermal circuit are illustrated as in Figure 6c,d. The process of heat conduction in this case can be regarded as heat transfer mainly in a parallel configuration of the thermal circuit. *R_1_*, *R*_2_, *R_H_*, and *R_conv_* represent the thermal resistances of the gypsum material, heat sink, as well as the thermal resistance for convection, respectively.

### 2.5. A Modified Thermal Circuit Including Transverse Thermal Resistance

**Figure 7:**
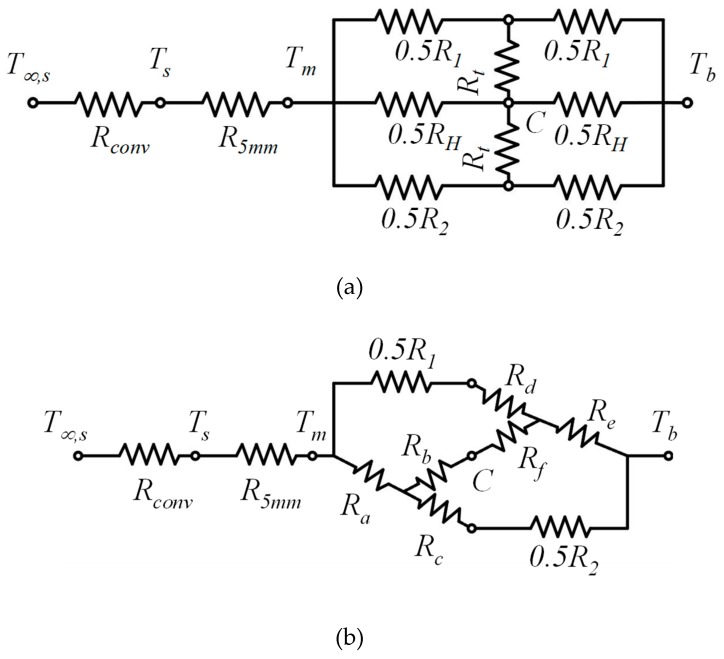
(**a**) The modified thermal circuit of the system including transverse thermal resistance, and (**b**) transformation of Δ and Y circuits for the circuit.

When the heat sink is too thick, it is inappropriate to assume that the heat diffusion is only in the *x*-direction, as shown in Figure 6c. In fact, the heat diffusion in the transverse direction (the *y*-direction) needs to be considered. A node *C* is added at the center of the heat diffusion path of the circuit shown in Figure 6d, and the temperature can be assumed to be the average of *T_m_* and *T_b_*. Two transverse thermal resistances *R_t_* are connected to both side paths of *R_1_* and *R*_2_ when considering transverse heat diffusion. The modified thermal circuit is sketched in Figure 7a.

Solving the modified thermal circuit problem, as shown in Figure 7a, the transformation of Δ and Y circuits [20] should be utilized to calculate the equivalent resistance of the circuit. Two Y-shaped resistance circuits are added into the upper right and the bottom left loops of the modified thermal circuit, and thus, the equivalent circuit is shown in Figure 7b. The resistances on the border of the loops are replaced by (*R_a_*, *R_b_*, *R_c_*) and (*R_d_*, *R_e_*, *R_f_*) and expressed by:

(15) $$R_{a}=\frac{0.25R_{2}R_{H}}{0.5\left(R_{2}+R_{H}\right)+R_{t}}$$

(16) $$R_{b}=\frac{0.5R_{H}R_{t}}{0.5\left(R_{2}+R_{H}\right)+R_{t}}$$

(17) $$R_{c}=\frac{0.5R_{t}R_{2}}{0.5\left(R_{2}+R_{H}\right)+R_{t}}$$

(18) $$R_{d}=\frac{0.5R_{t}R_{1}}{0.5\left(R_{1}+R_{H}\right)+R_{t}}$$

(19) $$R_{e}=\frac{0.25R_{1}R_{H}}{0.5\left(R_{1}+R_{H}\right)+R_{t}}$$

(20) $$R_{f}=\frac{0.5R_{H}R_{t}}{0.5\left(R_{1}+R_{H}\right)+R_{t}}$$

**Figure 8:**
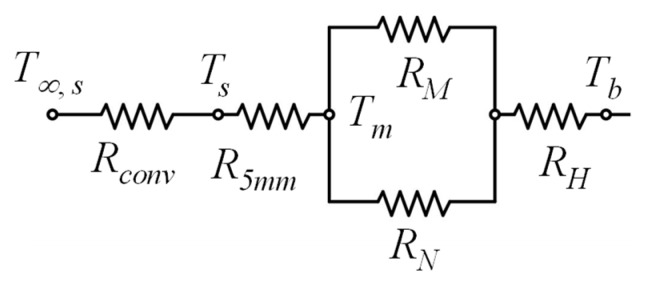
Modified thermal circuit including transverse thermal resistance.

Furthermore, a sequential calculation of the transformation skill of Δ and Y circuits is implemented, and finally, the circuit shown in Figure 7a is simplified and illustrated in Figure 8. The equivalent resistance of the modified thermal circuit with transverse thermal resistance can be determined by:

(21) $$R_{e}^{p}=R_{5mm}+{\left(\frac{1}{R_{M}}+\frac{1}{R_{N}}\right)}^{-1}+R_{H}+R_{conv}$$

where:

(22) $$\{\begin{array}{c} R_{M}=0.5R_{1}+R_{d}+\frac{R_{f}\left(R_{c}+R_{e}\right)}{R_{b}+R_{c}+R_{e}+R_{f}+0.5R_{2}}\\ R_{N}=R_{a}+R_{d}+\frac{\left(R_{b}+0.5R_{2}\right)\left(R_{c}+R_{e}\right)}{R_{b}+R_{c}+R_{e}+R_{f}+0.5R_{2}} \end{array}$$

Equations (21) and (22) provide a modified approach to treating the heat conduction of the gypsum insert, including the transverse heat diffusion.

### 2.6. Calculation of Mold Temperature Based on Thermal Circuit

Suppose that a gypsum insert is assembled into the mold base firmly. *T_s_* and *T_b_* are the cavity temperature and the bottom temperature of the gypsum insert. According to the methodology developed in the previous section, the theoretical mold temperature, *T_m_*, in a steady state can be calculated by:

(23) $$T_{m}=\left(\frac{R_{e}^{i}-R_{5mm}}{R_{e}^{i}}\right)T_{s}+\frac{R_{5mm}}{R_{e}^{i}}T_{b}\;i=s,\;p$$

where $R_{e}^{i}$ (*i* = *s* for a series configuration, *i* = *p* for a parallel configuration) is an equivalent resistance of the thermal circuit.

## 3. Experiment

### 3.1. Materials 

The gypsum material used in this study was calcium sulphate hemihydrate (*α*-type, CK25 from Kuang Pang Gypsum Ltd., Taiwan). When hemihydrate is mixed with water, it partially dissolves, and the solution is saturated with respect to Ca^2+^ and ${\mathrm{SO}}_{4}^{2-}$ ions. This saturated solution becomes supersaturated with respect to calcium sulphate dihydrate, leading to nucleation and crystal growth, and then, the strength of the insert is developed. The optimal ratio of hemihydrate to water for the gypsum insert is about 65–68 wt.%, and the detailed process of the preparation of a gypsum insert can be referred to in previous work [1]. The material of the heat sink used for the experiment was aluminum alloy (Al6061 by C. S. Aluminium Corporation, Taiwan). The thermal conductivities of steel, gypsum, and aluminum were 51, 0.5, and 237 W/m·K, respectively. The heat sink with dimensions of 60 × 60 mm in width and length, respectively, and three kinds of thicknesses, 10 mm, 20 mm, and 40 mm, was prepared. The plastic material used for injection molding was PP resin (polypropylene, GP-6331 from the LCY Chemical Corp., Taiwan) with a material property that can be found on the LCY Company’s website (www.lcygroup.com). In order to preventively reduce the effect of moisture in the molding process, the residual moisture that could exist in the package of PP material was reduced using a dehumidification process (2 h and 80 °C) before injection molding.

### 3.2. Test Mold and Temperature Measurement

**Figure 9:**
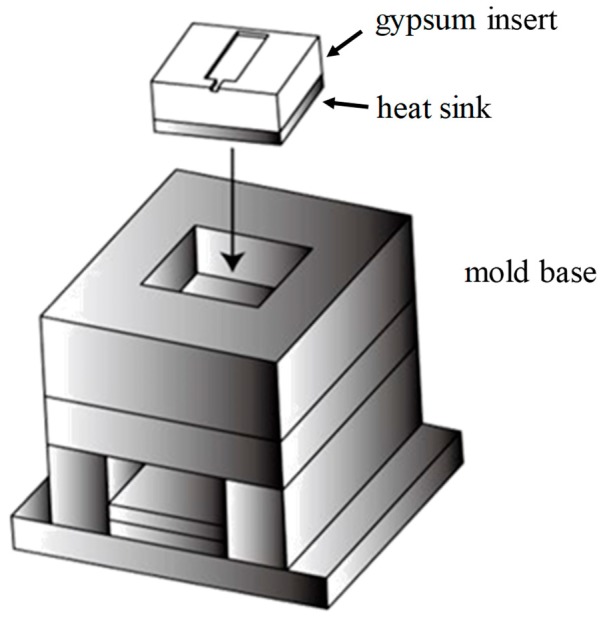
The gypsum insert and the mold base.

**Figure 10:**
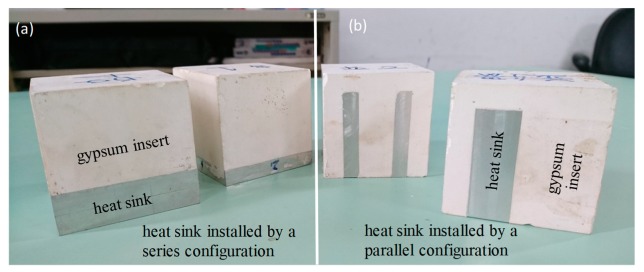
(**a**) a gypsum insert installed with a heat sink in accordance with a series configuration, (**b**) a gypsum insert installed with a heat sink in accordance with a parallel configuration.

The test mold consisted of a mold base made of steel (grade: S45C) and two mold inserts installed on each side of the mold halves. An illustration of the gypsum insert assembled in the mold base is shown in Figure 9. The cooling channel of the test mold was designed on the mold plate behind the insert. The dimensions of the gypsum insert were 60 mm × 60 mm × 63 mm for width, length, and height, respectively. The gypsum insert is formed by casting the gypsum paste into a box [1]. This process allows a heat sink to be easily installed into the gypsum insert. The inserts with the heat sink in a series or a parallel configuration are shown in Figure 10a,b. 

**Figure 11:**
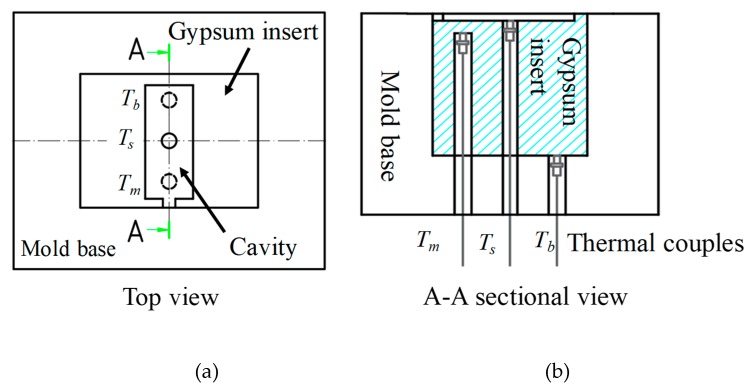
Three locations of the gypsum insert for temperature measurement, (**a**) top view of the mold and (**b**) A–A sectional view of three thermal couples.

Three sets of temperature data for the gypsum insert, *T*_s_, *T_m_*, and *T_b_*, were collected, and a sketch of the installation locations of the thermocouples is shown in Figure 11a,b. The cavity temperature *T*_s_ was measured at the cavity surface. The mold temperature *T_m_* was measured at a depth of 5 mm behind the cavity surface of the gypsum insert. The bottom temperature *T_b_* was measured at the bottom surface of the insert. A thermocouple (TC6-k, accuracy: +/− 0.1°C) and data recorder (HOBO UX120-006M) were provided by Onset Computer Corporation, USA. The recording frequency of the HOBO UX120-006M was 1 Hz.

### 3.3. Injection Molding Process

The experiment was implemented in an air-conditioned room with the temperature and humidity set at 27 ± 2 °C and 55–60%, respectively. The coefficient associated with the free convection heat from the surface of the gypsum insert to the air was 15 W/m^2^·K [18]. The molding experiments were performed on the injection molding machine, Fanuc Robotshot S-2000i 50A, Japan. The molding parameters are listed in Table 1. A low holding pressure was selected to reduce premature destruction on the gypsum insert. In addition, there was no cooling channel machined in the gypsum insert so that the setting’s cooling time was longer than that of conventional molds. The cycle time of each injection shot was 90 s, including an interval between each injection shot.

## 4. Results and Discussion

The first example was implemented to measure three temperatures of the mold, as well as to provide the necessary boundary conditions for solving the heat transfer equation in the gypsum mold. These calculated data were used in the next two examples for validation of the measured and predicted mold temperatures. Finally, the influence of the gypsum mold with/without a heat sink on the distortion of the molded part was discussed in the final example.

### 4.1. The Evolution of Mold Temperature of Gypsum Mold during Injection Molding Process

**Figure 12:**
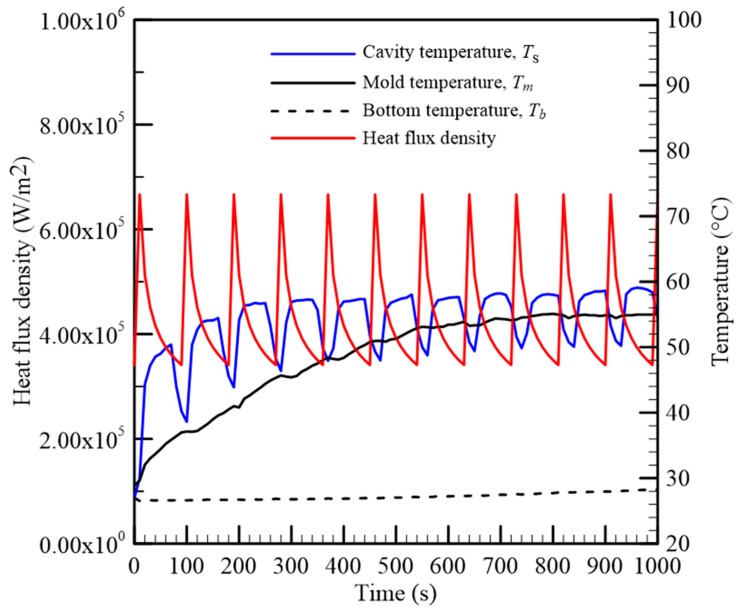
Cavity temperature, mold temperature, bottom temperature, and heat flux density of the gypsum mold in injection molding process.

The mold temperature is influenced by the heat of the molten polymer and the conduction capability of the mold. To provide sufficient boundary conditions for solving the heat transfer of the gypsum mold, the temperature at three locations in the gypsum insert, as mentioned in the previous section, needs to be measured in advance. After a series of injection molding experiments, those three temperatures and the heat flux density, with respect to time, are presented in Figure 12. In the filling stage of the injection process, the thermal shock of the molten polymer induced a significant heat flux density on the mold cavity. Therefore, the cavity temperature *T_s_* rose quickly to reach the maximum value, while the mold temperature *T_m_* was increased concurrently as the heat was delivered into the mold. *T_b_* was the temperature at the bottom of the gypsum mold. As the molten polymer was cooled by conduction, both temperatures, *T_s_* and *T_m_*, decreased. When the next shot started, the cavity temperature and the mold temperature were increased higher than those of the previous shot. Both of them behaved as a saw-tooth curve with respect to the injection shot. However, the fluctuation of the mold temperature curve was much less than that of the cavity temperature since the heat delivery of the gypsum material was poor. After 5–6 injection shots, the fluctuation amplitude of the mold temperature curve was reduced, approaching a plateau. The mold temperature was almost quasi-stable. Note that the comparison of the theoretical and experimental results was utilized in this stage.

In addition, these measured temperatures were used to calculate the coefficients *c*_1_ and *c*_0_ in Equation (8). In this study, we used the least square method to solve them, and the results were *c*_1_ = 5.62 and *c*_0_ = 12.52.

### 4.2. Effects of the Heat Sink Thickness and Installation Method (a Series Configuration) on the Mold Temperature

**Figure 13:**
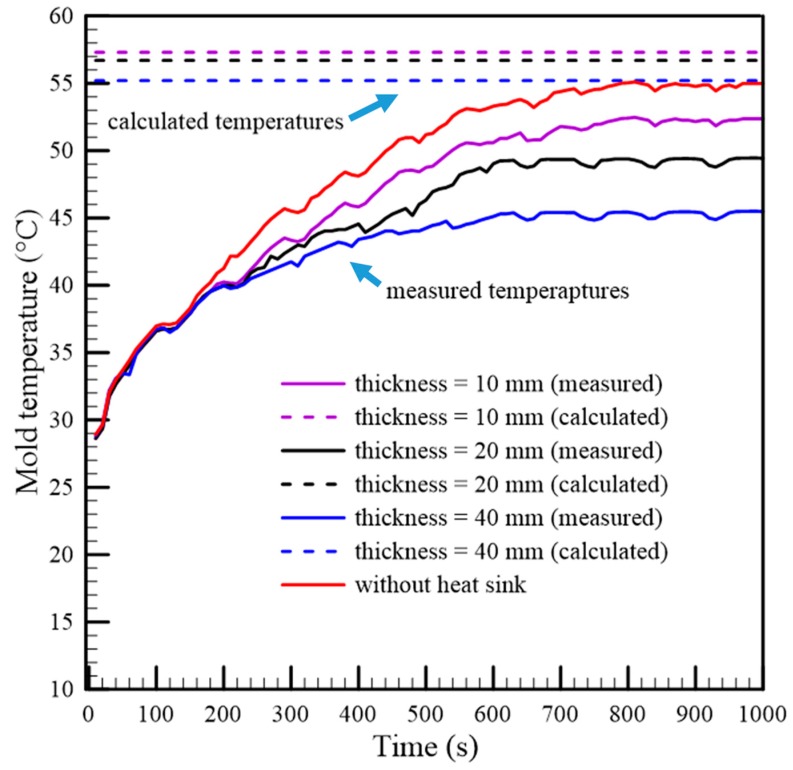
Comparison of the mold temperatures of the gypsum inserts in a series configuration.

Three gypsum inserts installed by using different heat sinks (thicknesses of 10 mm, 20 mm, and 40 mm) in accordance with a series configuration were prepared, individually. For theoretical analysis, the thermal circuit of the heat diffusion on the gypsum insert was constructed, as shown in Figure 5d. Each thermal resistance in the thermal circuit was calculated separately, and then, the equivalent thermal resistance $R_{e}^{s}$ and the heat transfer rate *q_x_* of the gypsum insert could be determined. Hence, the theoretical mold temperature *T*_m_ of each gypsum insert was calculated using Equation (23). In contrast with the calculated temperatures, those measured mold temperatures were collected using an injection molding experiment. Both the calculated and measured mold temperatures are listed in Table 2. The heat transfer rate was increased with respect to the increase in the heat sink thickness. A larger *q*_x_ of the thermal circuit meant that the heat diffusion efficiency in the insert was higher so that the mold temperature was lower. The calculated and the measured mold temperatures, with respect to time, are also shown in Figure 13. Compared to the gypsum insert without a heat sink, the maximum mold temperature reduction of the gypsum insert with an embedded heat sink of 40 mm thickness is 9.4 °C. The results also showed that the theoretical mold temperature was higher than the measured mold temperature. The root cause of deviation could be the simplified model that mainly considered the heat flow in the perpendicular direction and neglected some heat dissipation from the cavity surface when the mold opened. In addition, the temperature on each layer’s surface of the gypsum insert was mainly assumed to be the same. In fact, most of the heat from the molten polymer only accumulated in the cavity of the gypsum mold. However, the trend of the measured mold temperatures agreed with the theoretical results.

### 4.3. Effects of the Heat Sink Thickness and Installation Method (a Parallel Configuration) on the Mold Temperature 

**Figure 14:**
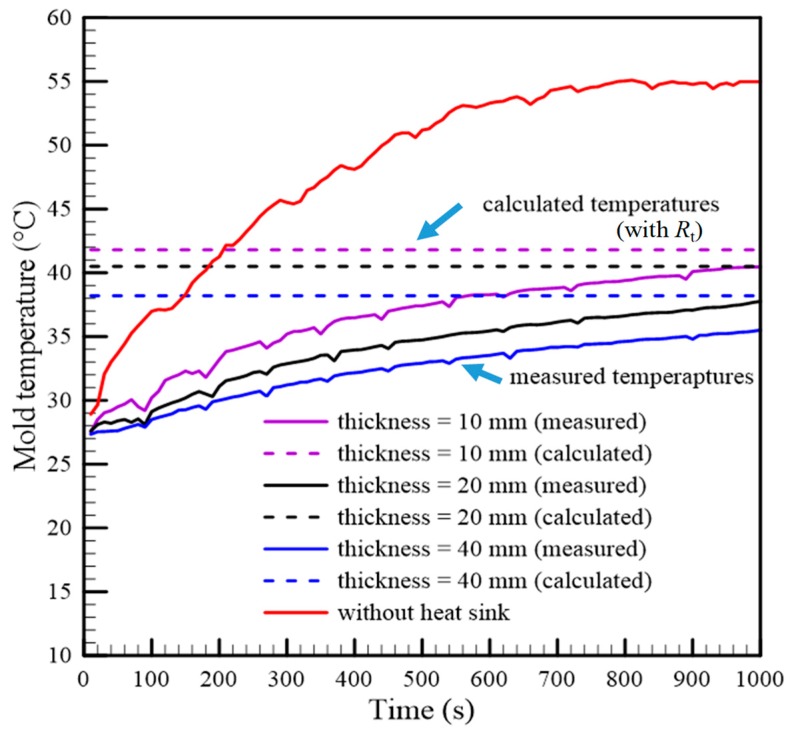
Comparison of the mold temperatures of the gypsum inserts in a parallel configuration.

**Figure 15:**
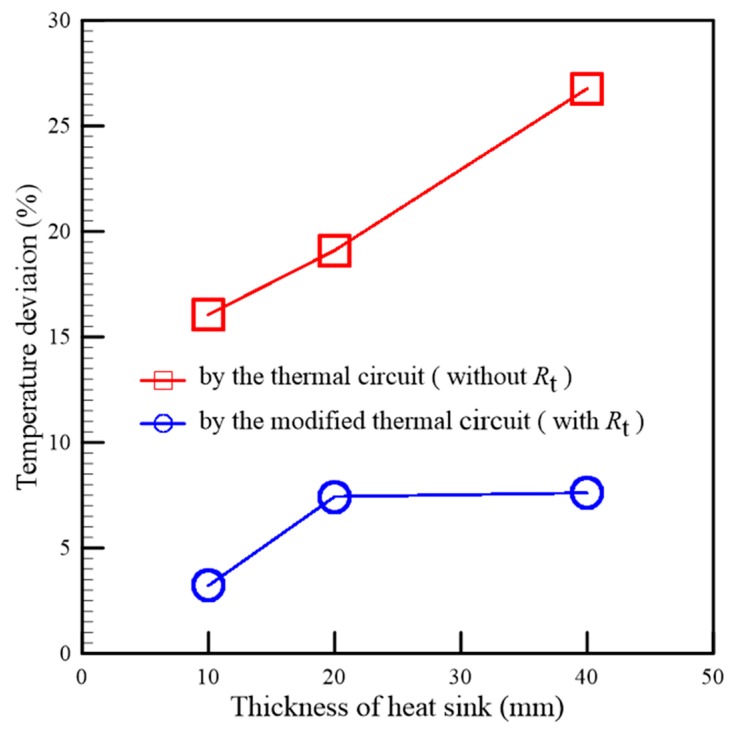
The accuracy of the mold temperature prediction by two different thermal circuits.

Three gypsum inserts installed using different heat sinks (thicknesses of 10 mm, 20 mm, and 40 mm) in accordance with a parallel configuration were prepared for temperature measurement. As the thermal circuit of each gypsum insert was constructed, with the heat transfer rate as determined first, the theoretical mold temperatures were calculated by using Equation (23). To investigate the effect of the transverse heat diffusion, their thermal circuits, including the transverse thermal resistance *R*_t_, were also constructed, respectively. In addition, a series of injection tests on the three gypsum inserts were implemented, and the measured and calculated mold temperatures are listed in Table 3. The curves of the calculated and measured mold temperatures, with respect to time, are also shown in Figure 14. Compared to Table 2, the heat transfer rates of the heat sink installation with a parallel configuration were larger than those of the series configuration installation. This meant that a higher efficiency of heat transfer was developed when the heat sink was installed in a parallel configuration. This also can be investigated using the slopes of the measured temperature curves which are less than those of the series configuration installation. The maximum mold temperature reduction of the proposed design in this example is 18.5 °C. The influence of the transverse thermal resistance on predicting the mold temperature is shown in Figure 15. The temperature deviation is defined by the ratio of the difference between the theoretical and measured mold temperatures to the measured temperature. The temperature deviation predicted by the thermal circuit without considering transverse thermal resistance *R*_t_ was larger than that predicted by the modified thermal circuit. This indicated that the mold temperature predicted by the thermal circuit without *R*_t_ was overestimated, and thus, the deviation between the calculated and the measured temperatures became large. It is worth noting that the temperature deviation increases as the heat sink thickness is increased if the transverse thermal resistance is not considered. 

### 4.4. Effect of Mold Temperature on the Deflection of the Molded Part

**Figure 16:**
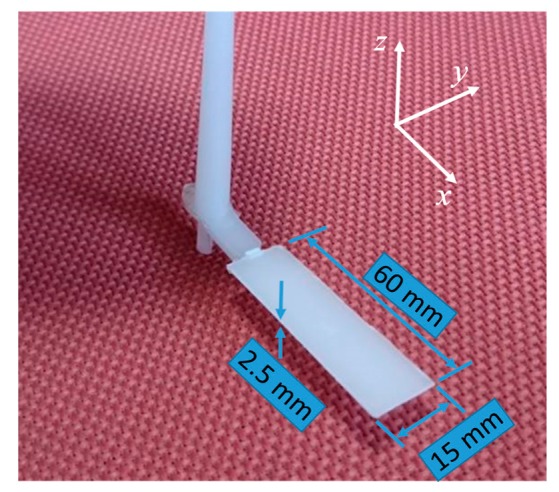
The molded part and its dimensions.

**Figure 17:**
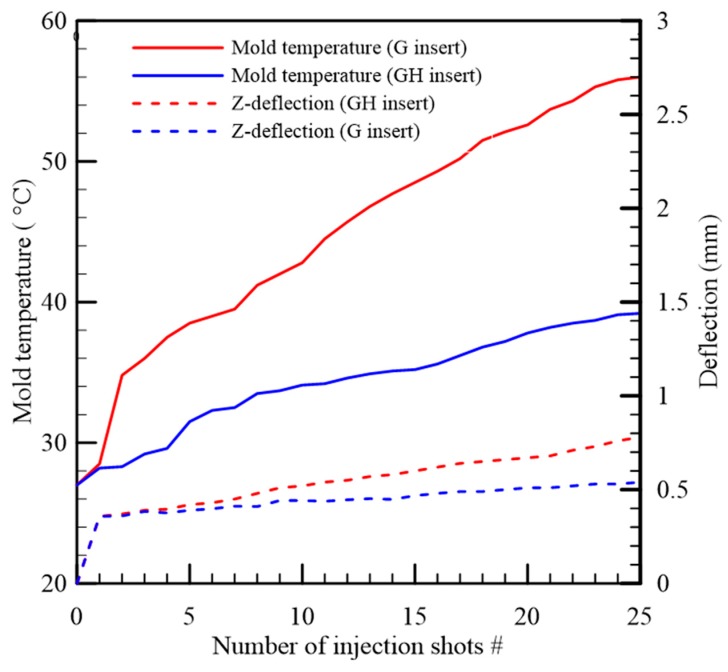
The mold temperature (referring to the left vertical axis) and deflection of the molded part (referring to the right vertical axis) with respect to the number of injection shots.

According to the previous example, we chose a gypsum insert installed with a 40 mm thick heat sink in a parallel configuration (GH insert) and a gypsum insert without any heat sink (G insert) for the experiment. The shape of the molded part, shown in Figure 16, was rectangular, and its dimensions were 60 mm × 15 mm × 2.5 mm in width, length, and thickness, respectively. The plastic material used for the experiment was PP (GP-6331). The *z*-directional deflection was a critical dimension to the part warpage, so we measured the distance between the lowest and the highest locations in the *z*-direction of the molded part. The molding parameters for both inserts were the same and are listed in Table 1, and the number of injection moldings for the measurement was 25 cycles. The molded parts were conditioned at 25 °C and 50% relative humidity for 24 h before dimensional measurement. The dimensional measurement was implemented using a coordinate measuring machine (Mitutoyo CRYSTA-Apex S544). The deflection of the molded part and the mold temperature with respect to each injection cycle are shown in Figure 17. Both of the solid lines represent the evolution of the measured mold temperatures of the GH insert and the G insert, respectively. The dashed lines are the deflections of the parts molded by the GH insert and the G insert, respectively. The increased rate of the mold temperature by the G insert was greater than that by the GH insert. It is well known that the higher the mold temperature, the greater the deflection of the molded part. In this study, the deflection of the molded part became more apparent after the mold temperature was over 50 °C. At the end of the test, the deflection of the final part molded by the GH insert was less than that of the G insert by 0.24 mm. This result demonstrated that the use of the heat sink made the mold temperature rise slowly and created a better molded part quality. In addition, the failures of both inserts were the crack in the cavity corner, and the lifetimes of the two inserts, the GH insert and the G insert, were 138 and 86 injection shots, respectively. 

## 5. Conclusions

A mold insert made of gypsum with an embedded heat sink to improve the mold temperature, has been demonstrated in this work. A methodology based on the thermal circuit concept was developed as well to provide a systematical approach to analyze the influences of the design parameters on the mold temperature. After the thermal resistances of all the components were determined either theoretically or experimentally, the thermal circuit of the mold was constructed, and then, the heat transfer rate was calculated. Based on this model, one could quickly gain the understanding of the physical essence behind the functionality of the heat sink. This helps us to determine quickly what design and engineering elements govern the mold temperature. In addition, we also provided a modified thermal circuit including the transverse heat transfer process and expanded the system to a 2D heat transfer model in this work. The results showed that the mold temperature was reduced more efficiently when the heat sink was embedded by a parallel configuration rather than by a series configuration. In this study, the mold temperature was reduced by 16.8 °C, while the deflection of the molded part was improved from 0.78 mm to 0.54 mm if the insert included a heat sink 40 mm thick in a parallel configuration. This method is very helpful to determine the arrangement of a cooling system at the very early design stages during the development of new molds. If detailed information is needed for further confirmation, the commercial simulation tool may be introduced cooperatively.

## Figures and Tables

**Table 1:** Molding parameters for the experiment.

Molding Parameter	
Barrel temperature, °C	205, 200, 190,180 (from Nozzle to Hopper)
Injection rate, cm^3^/s	17.1
Injection pressure, MPa	60
Holding pressure/time, MPa/s	10/2.0
Cooling time, s	60

**Table 2:** The calculated and the measured mold temperatures of the gypsum insert installed with a heat sink (in a series configuration).

Heat Sink Thickness mm	$R_{e}^{s}$ K/W	*q*_x_ W	*T*_m_ (Calculated) °C	*T*_m_ (Measured) °C
10	30.57	0.98	57.3	52.5, SD = 0.5
20	25.02	1.20	56.7	49.4, SD = 0.5
40	19.48	1.34	55.2	45.4, SD = 0.3

SD: standard deviation.

**Table 3:** Heat transfer rate and the calculated and measured mold temperatures in a parallel configuration of the gypsum insert.

Heat Sink Thickness mm	*q*_x_, W without *R_t_*	*q*_x_, W with *R_t_*	*T*_m_, °C (Calculated) without *R_t_*	*T*_m_, °C (Calculated) with *R_t_*	*T*_m_, °C (Measured)
10	9.8	7.54	34.0	41.8	40.5, SD = 0.4
20	11.04	8.74	30.5	40.5	37.7, SD = 0.4
40	14.75	9.52	26.0	38.2	35.5, SD = 0.2

*R_t_*: transverse thermal resistance. SD: standard deviation of the measured data.
